# 2,4-Di­chloro-*N*-[eth­yl(2-hy­droxy­eth­yl)carbamo­thio­yl]benzamide

**DOI:** 10.1107/S1600536813032881

**Published:** 2013-12-11

**Authors:** Bohari M Yamin, Suhaila Sapari, Siti Aishah Hasbullah

**Affiliations:** a School of Chemical Sciences and Food Technology, Universiti Kebangsaan Malaysia, 43600 Bangi, Selangor, Malaysia

## Abstract

In the title compound, C_12_H_14_Cl_2_N_2_O_2_S, the mol­ecule adopts a *cis* conformation with respect to the di­chloro­benzoyl group against the thiono group about the C—N bond. However, the di­chloro­benzene group and the thio­urea moiety are twisted by 75.41 (8)°. An intra­molecular N—H⋯O hydrogen bond occurs between the amido H atom and hydroxyl O atom. In the crystal, O—H⋯S and O—H⋯O hydrogen bonds link the molecules, forming chains along the *b-*axis direction.

## Related literature   

For bond-length data, see: Allen *et al.* (1987 ▶). For related structures of thio­urea derivatives, see: Hassan *et al.* (2010 ▶); Nasir *et al.* (2011 ▶); Al-abbasi *et al.* (2012 ▶); Yamin *et al.* (2013 ▶).
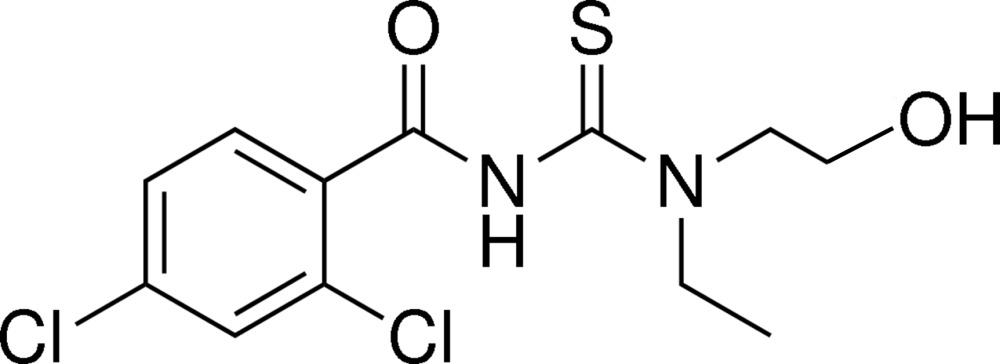


## Experimental   

### 

#### Crystal data  

C_12_H_14_Cl_2_N_2_O_2_S
*M* *_r_* = 321.21
Monoclinic, 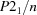

*a* = 6.9712 (4) Å
*b* = 10.6989 (6) Å
*c* = 19.3288 (10) Å
β = 96.441 (2)°
*V* = 1432.52 (14) Å^3^
*Z* = 4
Mo *K*α radiation
μ = 0.60 mm^−1^
*T* = 300 K
0.50 × 0.40 × 0.18 mm

#### Data collection  

Bruker SMART APEX CCD area-detector diffractometer
Absorption correction: multi-scan (*SADABS*; Bruker, 2009 ▶) *T*_min_ = 0.754, *T*_max_ = 0.900
30240 measured reflections
2967 independent reflections
2619 reflections with *I* > 2σ(*I*)
*R*_int_ = 0.036

#### Refinement  

*R*[*F* ^2^ > 2σ(*F* ^2^)] = 0.040
*wR*(*F* ^2^) = 0.126
*S* = 1.16
2967 reflections
176 parameters
1 restraint
H atoms treated by a mixture of independent and constrained refinement
Δρ_max_ = 0.44 e Å^−3^
Δρ_min_ = −0.36 e Å^−3^

### 

Data collection: *SMART* (Bruker,2009 ▶); cell refinement: *SAINT* (Bruker, 2009 ▶); data reduction: *SAINT*; program(s) used to solve structure: *SHELXTL* (Sheldrick, 2008 ▶); program(s) used to refine structure: *SHELXTL*; molecular graphics: *SHELXTL*; software used to prepare material for publication: *SHELXTL*, *PLATON* (Spek, 2009 ▶) and *publCIF* (Westrip, 2010 ▶).

## Supplementary Material

Crystal structure: contains datablock(s) global, I. DOI: 10.1107/S1600536813032881/rn2121sup1.cif

Structure factors: contains datablock(s) I. DOI: 10.1107/S1600536813032881/rn2121Isup2.hkl

Supporting information file. DOI: 10.1107/S1600536813032881/rn2121Isup3.cml

Additional supporting information:  crystallographic information; 3D view; checkCIF report

## Figures and Tables

**Table 1:** Hydrogen-bond geometry (Å, °)

*D*—H⋯*A*	*D*—H	H⋯*A*	*D*⋯*A*	*D*—H⋯*A*
N1—H1*A*⋯O2	0.86	1.99	2.796 (3)	155
O2—H2*A*⋯S1^i^	0.81 (3)	2.75 (3)	3.438 (2)	143 (3)
O2—H2*A*⋯O1^i^	0.81 (3)	2.25 (3)	2.920 (3)	140 (3)

Symmetry code: (i) 
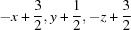
.

**Fig. 1.**
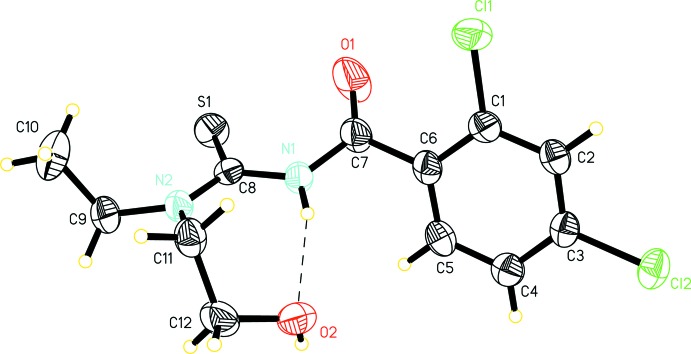
The molecular structure of (I), with displacement ellipsods drawn at the 50% probability level. The dashed line indicates the intramolecular hydrogen bond.

**Fig. 2.**
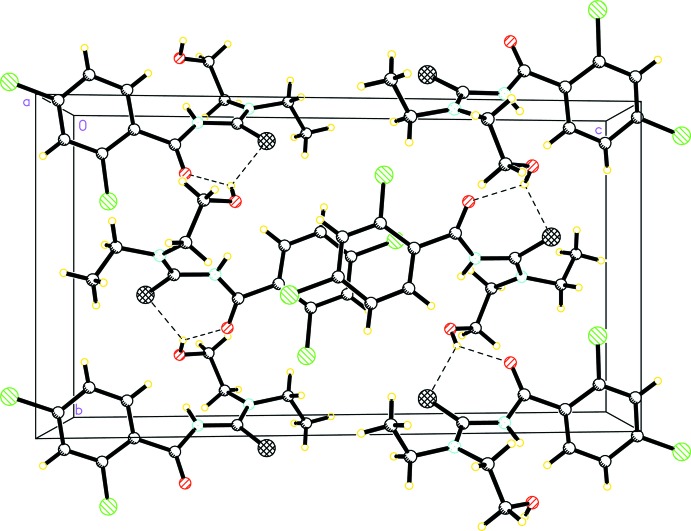
Molecular packing of (I) viewed down the a-axis. The dashed lines indicate intermolecular hydrogen bonds.

## supplementary crystallographic information

### 1. Comment

Most of the aroyl or carbonoyl thiourea compounds reported so far are based on primary amines. The two amino hydrogen atoms play an important role on the geometry of the thiourea moiety such as that in 3-chloro-N-[N-(furan-2-carbonyl)hydrazinocarbothioyl]benzamide (Yamin *et al.*, 2013) where it adopts trans geometry. However, in the secondary amine based thiourea, only the amido hydrogen atom is present (Nasir *et al.*, 2011; Al-abbasi *et al.*, 2012). Therefore, it can be expected that in the secondary amine carbonoyl thiourea, cis configuration is more likely to occur due to the absence of intrahydrogen bond involving the carbonyl oxygen atom and thioamide hydrogen atom. The title compound consists of dichloro substituted benzoyl and ethylethanol groups attached to the terminal nitrogen atoms respectively (Fig.1). The dichlorobenzoyl group is cis against thiono group across the C7—N1 bond. They are not coplanar but twisted by the dihedral angle between thiourea fragment S1/N1/N2/C8 and the dichlorobenzene ring, Cl1/Cl2/C1-C6, of 75.41 (8)°. Each fragment is planar with the maximum deviation of 0.025 (1)Å for atom Cl1 from the least-squares plane. The bond lengths and angles are in normal ranges (Allen *et al.*,1987). There is an intramolecular hydrogen bond N1–H1A···O2 between the amido hydrogen and hydroxyl oxygen atom. In the crystal structure, the molecules are linked by O2–H2A···S1 and O2–H2A···O1 intermolecular hydrogen bonds (see Table 1 for symmetry codes) to form one-dimensional chains along the b-axis (Fig.2).

### 2. Experimental

An acetone (30 ml) solution of (ethylamino)ethanol (0.18 g, 2 mmol) was added to a round-bottomed flask containing 2,4-dichlorobenzoyl isothiocyanate (0.58 g,2 mmol). The mixture was refluxed for 3h. After cooling the solution was filtered off and the filtrate was left to evaporate at room temperature. The solid formed was washed with water and cold ethanol. Crystals suitable for X-ray study were obtained by recrystallization from DMSO.

### 3. Refinement

All H atoms attached to C and N atoms were fixed geometrically and treated as riding with C—H= 0.93-0.97Å and N–H = 0.86Å with U_iso_(H)= 1.2U_eq_[C (methylene and aromatic),N] and 1.5 U_eq_ [C (methyl)]. The hydroxyl hydrogen atom was located from Fourier map and refined isotropically with O-H restraint to 0.82Å with an esd of 0.01.

### Crystal data

**Table:**

C_12_H_14_Cl_2_N_2_O_2_S	*F*(000) = 664
*M**_r_* = 321.21	*D*_x_ = 1.489 Mg m^−^^3^
Monoclinic, *P*2_1_/*n*	Mo *K*α radiation, λ = 0.71073 Å
Hall symbol: -P 2yn	Cell parameters from 18947 reflections
*a* = 6.9712 (4) Å	θ = 2.8–26.5°
*b* = 10.6989 (6) Å	µ = 0.60 mm^−^^1^
*c* = 19.3288 (10) Å	*T* = 300 K
β = 96.441 (2)°	Block, colourless
*V* = 1432.52 (14) Å^3^	0.50 × 0.40 × 0.18 mm
*Z* = 4	

### Data collection

**Table:**

Bruker SMART APEX CCD area-detector diffractometer	2967 independent reflections
Radiation source: fine-focus sealed tube	2619 reflections with *I* > 2σ(*I*)
Graphite monochromator	*R*_int_ = 0.036
Detector resolution: 83.66 pixels mm^-1^	θ_max_ = 26.5°, θ_min_ = 2.8°
ω scans	*h* = −8→8
Absorption correction: multi-scan (*SADABS*; Bruker, 2009)	*k* = −13→13
*T*_min_ = 0.754, *T*_max_ = 0.900	*l* = −24→24
30240 measured reflections	

### Refinement

**Table:**

Refinement on *F*^2^	Primary atom site location: structure-invariant direct methods
Least-squares matrix: full	Secondary atom site location: difference Fourier map
*R*[*F*^2^ > 2σ(*F*^2^)] = 0.040	Hydrogen site location: inferred from neighbouring sites
*wR*(*F*^2^) = 0.126	H atoms treated by a mixture of independent and constrained refinement
*S* = 1.16	*w* = 1/[σ^2^(*F*_o_^2^) + (0.0666*P*)^2^ + 0.6739*P*] where *P* = (*F*_o_^2^ + 2*F*_c_^2^)/3
2967 reflections	(Δ/σ)_max_ = 0.002
176 parameters	Δρ_max_ = 0.44 e Å^−^^3^
1 restraint	Δρ_min_ = −0.36 e Å^−^^3^

### Special details

**Table:**

Geometry. All esds (except the esd in the dihedral angle between two l.s. planes) are estimated using the full covariance matrix. The cell esds are taken into account individually in the estimation of esds in distances, angles and torsion angles; correlations between esds in cell parameters are only used when they are defined by crystal symmetry. An approximate (isotropic) treatment of cell esds is used for estimating esds involving l.s. planes.
Refinement. Refinement of F^2^ against ALL reflections. The weighted R-factor wR and goodness of fit S are based on F^2^, conventional R-factors R are based on F, with F set to zero for negative F^2^. The threshold expression of F^2^ > 2sigma(F^2^) is used only for calculating R-factors(gt) etc. and is not relevant to the choice of reflections for refinement. R-factors based on F^2^ are statistically about twice as large as those based on F, and R- factors based on ALL data will be even larger.

### Fractional atomic coordinates and isotropic or equivalent isotropic displacement parameters (Å^2^)

**Table:**

	*x*	*y*	*z*	*U*_iso_*/*U*_eq_	
Cl1	0.89241 (10)	0.71322 (6)	0.93151 (4)	0.0554 (2)	
Cl2	0.70111 (8)	1.08652 (6)	1.09352 (3)	0.04474 (18)	
S1	0.75350 (9)	0.90835 (6)	0.64182 (3)	0.04555 (19)	
O1	0.7295 (4)	0.8000 (2)	0.78711 (10)	0.0845 (9)	
O2	1.0855 (3)	1.20198 (15)	0.81005 (9)	0.0448 (4)	
N1	0.9083 (3)	0.97467 (17)	0.77030 (9)	0.0367 (4)	
H1A	0.9742	1.0316	0.7937	0.044*	
N2	1.0766 (3)	1.03352 (17)	0.68075 (9)	0.0336 (4)	
C1	0.8163 (3)	0.8659 (2)	0.93868 (12)	0.0347 (5)	
C2	0.7904 (3)	0.9090 (2)	1.00426 (11)	0.0341 (5)	
H2B	0.8090	0.8564	1.0427	0.041*	
C3	0.7364 (3)	1.0320 (2)	1.01142 (11)	0.0331 (4)	
C4	0.7097 (3)	1.1128 (2)	0.95552 (12)	0.0383 (5)	
H4A	0.6744	1.1956	0.9616	0.046*	
C5	0.7369 (3)	1.0677 (2)	0.89040 (12)	0.0389 (5)	
H5A	0.7198	1.1211	0.8523	0.047*	
C6	0.7895 (3)	0.9437 (2)	0.88045 (11)	0.0348 (5)	
C7	0.8036 (4)	0.8964 (2)	0.80809 (12)	0.0446 (6)	
C8	0.9215 (3)	0.97372 (19)	0.69876 (11)	0.0322 (4)	
C9	1.0983 (4)	1.0593 (2)	0.60741 (11)	0.0422 (5)	
H9A	1.1651	1.1382	0.6043	0.051*	
H9B	0.9712	1.0677	0.5817	0.051*	
C10	1.2073 (4)	0.9592 (3)	0.57421 (14)	0.0611 (8)	
H10A	1.2171	0.9808	0.5265	0.092*	
H10B	1.1404	0.8811	0.5761	0.092*	
H10C	1.3344	0.9516	0.5987	0.092*	
C11	1.2412 (3)	1.0729 (2)	0.73058 (12)	0.0409 (5)	
H11A	1.3576	1.0700	0.7074	0.049*	
H11B	1.2566	1.0134	0.7687	0.049*	
C12	1.2214 (4)	1.2026 (2)	0.76032 (14)	0.0524 (6)	
H12A	1.3459	1.2306	0.7824	0.063*	
H12B	1.1792	1.2604	0.7230	0.063*	
H2A	1.000 (4)	1.253 (3)	0.8004 (19)	0.081 (12)*	

### Atomic displacement parameters (Å^2^)

**Table:**

	*U*^11^	*U*^22^	*U*^33^	*U*^12^	*U*^13^	*U*^23^
Cl1	0.0691 (4)	0.0333 (3)	0.0679 (4)	−0.0079 (3)	0.0262 (3)	−0.0030 (3)
Cl2	0.0400 (3)	0.0580 (4)	0.0379 (3)	−0.0032 (2)	0.0114 (2)	−0.0079 (2)
S1	0.0486 (4)	0.0481 (4)	0.0379 (3)	−0.0119 (3)	−0.0042 (3)	−0.0043 (2)
O1	0.134 (2)	0.0746 (15)	0.0452 (11)	−0.0699 (15)	0.0110 (12)	−0.0104 (10)
O2	0.0520 (10)	0.0357 (9)	0.0459 (9)	0.0000 (7)	0.0017 (8)	−0.0053 (7)
N1	0.0424 (10)	0.0378 (10)	0.0308 (9)	−0.0154 (8)	0.0083 (7)	−0.0074 (7)
N2	0.0371 (9)	0.0357 (9)	0.0283 (9)	−0.0033 (7)	0.0053 (7)	−0.0009 (7)
C1	0.0263 (9)	0.0346 (11)	0.0446 (12)	−0.0094 (8)	0.0101 (8)	−0.0020 (9)
C2	0.0248 (9)	0.0414 (12)	0.0366 (11)	−0.0071 (8)	0.0059 (8)	0.0043 (9)
C3	0.0216 (9)	0.0454 (12)	0.0329 (10)	−0.0039 (8)	0.0061 (7)	−0.0021 (9)
C4	0.0310 (10)	0.0399 (12)	0.0451 (12)	0.0016 (9)	0.0090 (9)	0.0016 (9)
C5	0.0321 (11)	0.0462 (13)	0.0390 (12)	−0.0038 (9)	0.0065 (9)	0.0078 (9)
C6	0.0275 (9)	0.0429 (12)	0.0349 (11)	−0.0120 (8)	0.0072 (8)	−0.0015 (9)
C7	0.0516 (13)	0.0450 (13)	0.0378 (12)	−0.0214 (11)	0.0077 (10)	−0.0044 (10)
C8	0.0383 (11)	0.0268 (10)	0.0316 (10)	0.0000 (8)	0.0047 (8)	−0.0035 (8)
C9	0.0478 (13)	0.0489 (13)	0.0306 (11)	−0.0012 (10)	0.0070 (9)	0.0062 (9)
C10	0.0576 (16)	0.087 (2)	0.0398 (13)	0.0117 (15)	0.0123 (12)	−0.0085 (14)
C11	0.0362 (11)	0.0508 (13)	0.0362 (11)	−0.0079 (10)	0.0064 (9)	−0.0002 (10)
C12	0.0644 (16)	0.0461 (14)	0.0464 (14)	−0.0239 (12)	0.0055 (12)	−0.0024 (11)

### Geometric parameters (Å, º)

**Table:**

Cl1—C1	1.728 (2)	C4—C5	1.381 (3)
Cl2—C3	1.734 (2)	C4—H4A	0.9300
S1—C8	1.668 (2)	C5—C6	1.395 (3)
O1—C7	1.203 (3)	C5—H5A	0.9300
O2—C12	1.423 (3)	C6—C7	1.501 (3)
O2—H2A	0.815 (10)	C9—C10	1.498 (4)
N1—C7	1.374 (3)	C9—H9A	0.9700
N1—C8	1.396 (3)	C9—H9B	0.9700
N1—H1A	0.8600	C10—H10A	0.9600
N2—C8	1.336 (3)	C10—H10B	0.9600
N2—C9	1.468 (3)	C10—H10C	0.9600
N2—C11	1.474 (3)	C11—C12	1.514 (4)
C1—C2	1.380 (3)	C11—H11A	0.9700
C1—C6	1.395 (3)	C11—H11B	0.9700
C2—C3	1.380 (3)	C12—H12A	0.9700
C2—H2B	0.9300	C12—H12B	0.9700
C3—C4	1.380 (3)		
			
C12—O2—H2A	112 (3)	N2—C8—N1	113.57 (17)
C7—N1—C8	128.33 (18)	N2—C8—S1	123.90 (16)
C7—N1—H1A	115.8	N1—C8—S1	122.48 (16)
C8—N1—H1A	115.8	N2—C9—C10	113.0 (2)
C8—N2—C9	121.02 (18)	N2—C9—H9A	109.0
C8—N2—C11	124.05 (17)	C10—C9—H9A	109.0
C9—N2—C11	114.86 (17)	N2—C9—H9B	109.0
C2—C1—C6	121.5 (2)	C10—C9—H9B	109.0
C2—C1—Cl1	117.57 (17)	H9A—C9—H9B	107.8
C6—C1—Cl1	120.91 (17)	C9—C10—H10A	109.5
C1—C2—C3	118.5 (2)	C9—C10—H10B	109.5
C1—C2—H2B	120.8	H10A—C10—H10B	109.5
C3—C2—H2B	120.8	C9—C10—H10C	109.5
C2—C3—C4	122.2 (2)	H10A—C10—H10C	109.5
C2—C3—Cl2	118.74 (16)	H10B—C10—H10C	109.5
C4—C3—Cl2	119.03 (17)	N2—C11—C12	114.4 (2)
C3—C4—C5	118.3 (2)	N2—C11—H11A	108.7
C3—C4—H4A	120.8	C12—C11—H11A	108.7
C5—C4—H4A	120.8	N2—C11—H11B	108.7
C4—C5—C6	121.6 (2)	C12—C11—H11B	108.7
C4—C5—H5A	119.2	H11A—C11—H11B	107.6
C6—C5—H5A	119.2	O2—C12—C11	110.37 (19)
C1—C6—C5	118.0 (2)	O2—C12—H12A	109.6
C1—C6—C7	122.3 (2)	C11—C12—H12A	109.6
C5—C6—C7	119.6 (2)	O2—C12—H12B	109.6
O1—C7—N1	125.2 (2)	C11—C12—H12B	109.6
O1—C7—C6	122.2 (2)	H12A—C12—H12B	108.1
N1—C7—C6	112.59 (18)		
			
C6—C1—C2—C3	0.2 (3)	C1—C6—C7—O1	44.6 (4)
Cl1—C1—C2—C3	177.72 (15)	C5—C6—C7—O1	−131.6 (3)
C1—C2—C3—C4	−0.7 (3)	C1—C6—C7—N1	−135.5 (2)
C1—C2—C3—Cl2	179.34 (15)	C5—C6—C7—N1	48.4 (3)
C2—C3—C4—C5	0.6 (3)	C9—N2—C8—N1	−170.21 (19)
Cl2—C3—C4—C5	−179.50 (16)	C11—N2—C8—N1	12.9 (3)
C3—C4—C5—C6	0.2 (3)	C9—N2—C8—S1	7.5 (3)
C2—C1—C6—C5	0.5 (3)	C11—N2—C8—S1	−169.33 (17)
Cl1—C1—C6—C5	−176.96 (15)	C7—N1—C8—N2	−160.2 (2)
C2—C1—C6—C7	−175.70 (19)	C7—N1—C8—S1	22.0 (3)
Cl1—C1—C6—C7	6.8 (3)	C8—N2—C9—C10	−92.3 (3)
C4—C5—C6—C1	−0.7 (3)	C11—N2—C9—C10	84.8 (3)
C4—C5—C6—C7	175.6 (2)	C8—N2—C11—C12	−89.9 (3)
C8—N1—C7—O1	13.0 (5)	C9—N2—C11—C12	93.1 (2)
C8—N1—C7—C6	−167.0 (2)	N2—C11—C12—O2	74.2 (3)

### Hydrogen-bond geometry (Å, º)

**Table:**

*D*—H···*A*	*D*—H	H···*A*	*D*···*A*	*D*—H···*A*
N1—H1*A*···O2	0.86	1.99	2.796 (3)	155
C9—H9*B*···S1	0.97	2.64	3.031 (3)	105
O2—H2*A*···S1^i^	0.81 (3)	2.75 (3)	3.438 (2)	143 (3)
O2—H2*A*···O1^i^	0.81 (3)	2.25 (3)	2.920 (3)	140 (3)

Symmetry code: (i) −*x*+3/2, *y*+1/2, −*z*+3/2.
